# Digital health platforms for intrinsic capacity monitoring in community-based integrated care for older adults: a scoping review

**DOI:** 10.3389/fpubh.2026.1847814

**Published:** 2026-08-05

**Authors:** Jundan Luo, Kai Bai, Jiaxin Sun, Zhiwen Deng, Chunlong Guo, Weidong Huang

**Affiliations:** School of Nursing, Changchun University of Chinese Medicine, Changchun, Jilin, China

**Keywords:** community, digital health platform, integrated care, intrinsic capacity, monitoring, older people

## Abstract

**Background:**

Integrated care has been advanced by the World Health Organization as a strategic response to population ageing. Digital health platforms are increasingly central to this agenda, particularly for monitoring intrinsic capacity among older adults within community-based integrated care. This scoping review synthesizes the implementation and practice evidence on digital health platforms used to monitor intrinsic capacity in this population.

**Methods:**

This review followed the methodological framework proposed by Arksey and O’Malley. Six databases—PubMed, Web of Science, the Cochrane Library, IEEE Xplore, Wiley Online Library and ProQuest—were systematically searched for studies published between 1 January 2000 and 31 December 2025. Two reviewers independently screened eligible studies and extracted data. Study characteristics and key features of the digital health platforms were narratively synthesized.

**Results:**

Fifteen studies involving 11 digital health platforms were included. Core platform functions comprised routine monitoring, risk alerts, multidimensional assessment and health reminders. Data sources included wearable devices, environmental sensors, questionnaires, user-entered information and medical devices. Overall, digital health platforms showed potential to improve older adults’ self-management capacity, health status and efficiency of health-care resource utilization, although findings varied substantially across studies. Barriers and facilitators to implementation were grouped into four domains: older users, caregivers, health-care providers and platform technology.

**Conclusion:**

Digital health platform–enabled integrated care can support continuous, dynamic monitoring of intrinsic capacity and promote proactive health management among community-dwelling older adults. Successful implementation, however, requires coordinated action across older users, caregivers, health-care professionals and technology systems. Future efforts should strengthen user training and technical support, expand caregiver-support functions, enhance interoperability with health-care systems, and improve device compatibility and accessibility. These strategies may accelerate the scalable adoption of digital health platforms in community-based integrated care for older populations.

## 1 Introduction

Global population ageing has evolved from a regionally confined phenomenon into a universal challenge confronting all societies. Between 2000 and 2050, the proportion of individuals aged 60 years and older is projected to double from 11 to 22% (1). This demographic transition is particularly pronounced in Asia and Eastern Europe. At present, European countries exhibit the highest proportion of older adults relative to total population, whereas Asian countries account for the largest absolute number of older individuals; several Asian nations have already entered a “super-aged society,” in which individuals aged 65 years and above comprise more than 20% of the population (2, 3). These shifts in population structure necessitate profound adaptations in both public health systems and socioeconomic frameworks.

On the one hand, cumulative physiological decline renders older adults increasingly susceptible to multimorbidity and complex health conditions, placing substantial demands on healthcare systems. On the other hand, the resulting long-term care needs impose significant socioeconomic burdens and generate considerable strain on informal family caregivers (4). However, traditional healthcare systems—largely designed around acute, single-disease management—are ill-equipped to address the multifaceted needs of older adults characterized by frailty and chronic multimorbidity.

Against this backdrop, the World Health Organization (WHO) has introduced the Integrated Care for Older People (ICOPE) framework, a person-centred and evidence-informed strategy designed to address the challenges of population ageing (5). At its core, ICOPE proposes an integrated care pathway that systematically screens, assesses, and manages declines in intrinsic capacity (6). Intrinsic capacity refers to the composite of an individual’s physical and mental capacities, encompassing five key domains: cognition, locomotion, vitality, sensory function, and psychological well-being. Functional ability, in turn, represents the interaction between intrinsic capacity and the surrounding environment, and is a critical determinant of healthy ageing (7–9). A key challenge for large-scale implementation of ICOPE in community settings lies in achieving cost-effective, accessible, continuous, and standardized monitoring of intrinsic capacity.

In recent years, rapid advances in digital health technologies—including the Internet of Things (IoT), mobile health, big data analytics, and artificial intelligence—have provided transformative tools to address these challenges. Digital health platforms, encompassing wearable devices, ambient sensors, smartphone applications, and remote monitoring systems, enable continuous and objective collection of physiological parameters, behavioral patterns, and functional status in older adults (10–13). For example, smartwatches can track gait speed and functional performance (14), interactive tablet-based applications can support cognitive assessment and training (15), and home-based sensors can capture daily activity trajectories (16). Collectively, these technologies enable remote, continuous, and objective monitoring of functional and behavioral changes in daily life, thereby facilitating remote screening and assessment, early identification of intrinsic capacity decline, personalized intervention delivery, and reduced workload for community healthcare providers (17).

Despite these promising developments, current research and implementation of digital health platforms for monitoring intrinsic capacity within community-based integrated care remain fragmented. Existing platforms differ substantially in their technological architectures, the intrinsic capacity domains they target, and the extent to which they are integrated into existing community care services. Moreover, their real-world effectiveness and implementation barriers have not yet been systematically synthesized. Therefore, this review aims to identify the facilitators and barriers associated with the use of digital health platforms for monitoring intrinsic capacity in community-based integrated care for older adults, thereby providing evidence to inform technology developers, healthcare providers, policymakers, and researchers.

## 2 Methods

This study was conducted in accordance with the PRISMA-ScR reporting guidelines (18) and aimed to systematically identify the implementation barriers and facilitators associated with digital health platforms for monitoring intrinsic capacity within community-based integrated care for older adults (19). A scoping review methodology was employed, guided by the framework originally proposed by Arksey and O’Malley (20) and subsequently refined by Levac et al. (21).

### 2.1 Stage 1: identifying the research questions

The study aimed to systematically review the existing evidence on digital health platforms used to monitor intrinsic capacity in community-based integrated care for older adults, including their technological implementations, preliminary outcomes, and the implementation barriers and facilitators encountered during deployment. To define the scope, the research team conducted an initial literature scoping and employed the PCC framework (Population, Concept, Context) (22), which informed the formulation of the following research questions:

1. What digital health platforms are currently available for monitoring intrinsic capacity in community-dwelling older adults receiving integrated care, and what are their core functionalities?
2. What are the reported preliminary outcomes of these digital health platforms?
3. What barriers and facilitators influence their implementation and integration into practice?

### 2.2 Stage 2: identifying relevant studies

A comprehensive systematic search was conducted across PubMed, Web of Science Core Collection, The Cochrane Library, IEEE Xplore, Wiley Online Library, and ProQuest, covering studies published between 1 January 2000 and 31 December 2025. The search strategy was developed in accordance with the PCC framework: Population (P) comprised community-dwelling older adults; Concept (C) focused on digital health platforms and intrinsic capacity monitoring; and Context (Co) referred to integrated care settings.

Search terms combined controlled vocabulary and free-text keywords, encompassing geriatric populations, community living, wearable devices, mobile applications, remote monitoring, artificial intelligence, and domains of intrinsic capacity including cognition, locomotion, vitality, sensory function, and psychological well-being, as well as integrated care, comprehensive care, person-centred care, and multidisciplinary collaboration.

The PubMed search strategy, as an example, was constructed as follows:

((((((aged[MeSH Terms]) OR (older adults[MeSH Terms])) OR (senior)) OR (older people[MeSH Terms])) AND ((((community-dwelling) OR (community)) OR (aging in place)) OR (independent living))) AND (((((intrinsic capacity) OR (functional capacity)) OR (frailty)) OR (physical function)) OR (cognitive function))) AND (((((((((((digital health[MeSH Terms]) OR (telemedicine[MeSH Terms])) OR (mobile applications[MeSH Terms])) OR (wearable electronic devices[MeSH Terms])) OR (eHealth)) OR (mHealth)) OR (telehealth)) OR (remote monitoring)) OR (mobile app)) OR (sensors)) OR (artificial intelligence)).

### 2.3 Stage 3: study selection

All retrieved records were imported into EndNote X9 for reference management and duplicate removal. The screening process was independently conducted by two reviewers in three sequential stages. In the initial screening phase, titles and abstracts of all retrieved records were independently assessed against the eligibility criteria, and clearly irrelevant studies were excluded. In the full-text screening phase, the complete texts of all preliminarily included articles were retrieved and independently evaluated by both reviewers to determine final eligibility for inclusion. Any discrepancies between the two reviewers were resolved through discussion and consensus. When disagreement persisted, a third senior reviewer adjudicated the final decision to determine the definitive study set.

#### 2.3.1 Inclusion criteria

(1) participants aged ≥60 years; (2) studies conducted in community or home-based settings; (3) use of digital health platforms to monitor at least one domain of intrinsic capacity as defined by the World Health Organization (i.e., cognition, mobility, vitality, sensory function, or psychological capacity); (4) empirical study designs; (5) published in English.

#### 2.3.2 Exclusion criteria

(1) Editorials, commentaries, or systematic reviews; (2) non-human studies; (3) participants aged <60 years; (4) intervention protocols only, without reporting actual study findings; (5) studies conducted exclusively in institutional settings (e.g., hospitals or rehabilitation centers); (6) full text unavailable.

### 2.4 Stage 4: charting the data

Data were independently extracted by two reviewers using a standardized data extraction form, including the following variables: first author, publication year, country, study design, study population, sample size, intervention duration, platform name, user interface characteristics, core functionalities, intrinsic capacity domains monitored, data sources, and reported outcome measures. Any discrepancies between reviewers were resolved through discussion and consensus.

### 2.5 Stage 5: collating, summarizing, and reporting results

**Figure 1:**
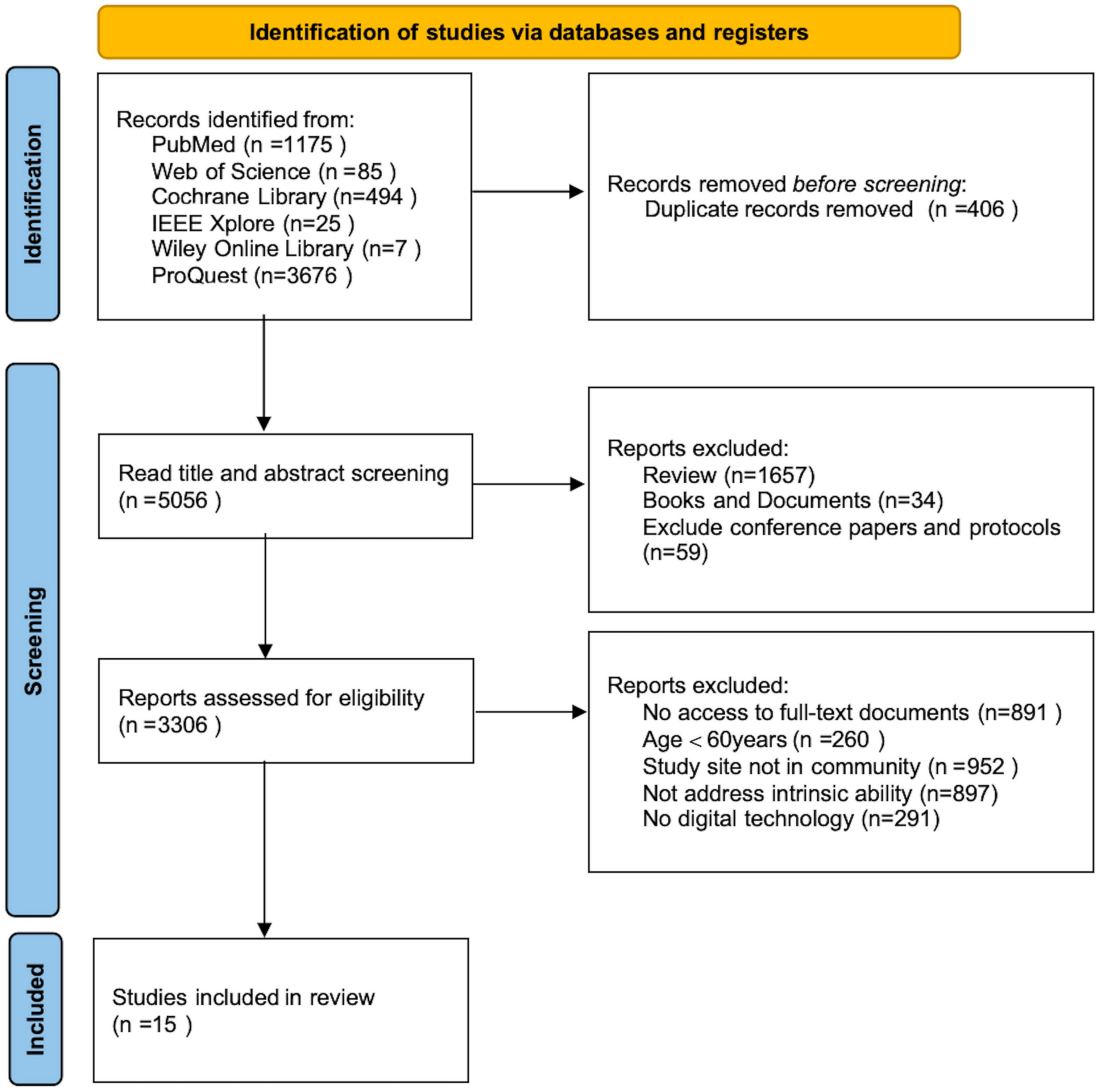
Flowchart of the literature screening process.

The study selection process and results were presented in a PRISMA flow diagram, detailing the number of records identified from each database, the number of records after deduplication, the outcomes of title and abstract screening, the results of full-text eligibility assessment, and the final number of included studies, along with reasons for exclusion at each stage (Figure 1).

## 3 Results

### 3.1 Search results

A total of 5,462 records were identified through database searching. After removing duplicates (*n* = 406), 5,056 records underwent title and abstract screening. Following full-text assessment, 15 studies were ultimately included in the final analysis.

The included studies comprised one randomized controlled trial (23), five prospective cohort studies (24–28), two cross-sectional studies (29, 30), three descriptive studies (31–33), two qualitative studies (34, 35), one mixed-methods study (36), and one observational study (37). Geographically, the studies were conducted in Europe (*n* = 10; 66.67%) (24–28, 30, 31, 35–37) and Asia (*n* = 5; 33.33%) (23, 29, 32–34).

Digital health–enabled integrated care models primarily targeted community-dwelling older adults (*n* = 10; 66.67%) (23, 24, 27, 29–34, 36), older adults with multimorbidity (*n* = 4; 17.39%) (25, 26, 28, 35), populations involving both caregivers and healthcare professionals (*n* = 3; 13.04%) (24, 25, 33), and individuals living alone (*n* = 2; 8.3%) (32, 37).

### 3.2 Key findings

This study encompassed 11 digital health platforms, which were systematically analyzed with respect to their technical characteristics, user access devices, core functionalities, integrated care monitoring domains, data sources, and reported application outcomes.

#### 3.2.1 Types and core functionalities of digital health platforms

Among the 15 included studies, 11 distinct digital health platforms were identified for intrinsic capacity monitoring in older adults. Six platforms assessed all five domains of intrinsic capacity (24, 27, 30, 31, 33, 34, 36), namely locomotion, psychological capacity, vitality, cognition, and sensory function.

The CAREUP platform (24) integrates core functions including daily monitoring, data sharing, anomaly detection, health management, interactive cognitive games, personalized care planning, and machine-learning–based predictive analytics. The ICOPE remote monitoring platform (27, 31) provides daily monitoring, comprehensive assessment, data sharing, alert systems, and referral or follow-up functionalities. Building upon the CAREUP framework (24), the DHS-ICM system (34) additionally incorporates comprehensive assessment, health reminders, emotional support, and digital avatar–based interaction. The iHealth Screen platform (33) offers daily monitoring, comprehensive assessment, and a resource center. Although the ICOPE mobile application (30) belongs to the same digital ecosystem as the ICOPE remote monitoring platform (27, 31), it is functionally more limited, providing only assessment and alerting capabilities. The SHAPES platform (36) extends beyond daily monitoring, data sharing, alerts, and machine-learning prediction by incorporating user need profiling and the delivery of personalized care services. Detailed characteristics are presented in Table 1.

**Table 1:** Summary of digital health platform characteristics.

Author, year	Country	Study design	Sample size/intervention duration	Platform name	Core functions	Outcomes
Kolakowski et al. (2025) (24)	Austria, Italy, Romania	Prospective cohort study	64/not specified	CAREUP	Routine monitoring, Cognitive game interaction, Data sharing hub, Machine learning prediction, Personalized care/goal plans, Abnormal alerts, Health management.	*Enabling factors:* The platform is user-friendly and effectively monitors health status, prevents falls, and improves cognitive function; the predictive model achieved an MAE of ≤ 0.14 across all IC domains, indicating acceptable performance. *Major barriers:* The cost of the platform may place a financial burden on older adults; usage frequency is low; predictive accuracy in the psychological domain is low.
Tavassoli et al. (2021) (31)	France	Descriptive study	950/assessments every 4–6 months	ICOPE Remote Monitoring Platform	Routine monitoring, Comprehensive assessment, Data sharing hub, Abnormal alerts, Referral/follow‑up	*Enabling factors:* preliminary data indicate that 92.6% of the older participants surveyed have impaired function in at least one domain; the platform empowers older adults to take an active role in managing their own health, and the system enables large-scale remote monitoring and timely intervention. *Major barriers*: intermittent monitoring may fail to detect acute changes in health status.
Tavassoli et al. (2022) (27)	France	Prospective cohort study	10,903/minimum 6-month follow-up			*Enabling factors*:94.3% of participants had potential decline in at least one capability domain. Among users who self‑assessed with digital tools, 78.2% expressed satisfaction with the tools. *Major barriers*: a high screening positivity rate may induce anxiety; some older adults rely on others’ help to use the tools; a lack of compensation mechanisms leads to low participation among healthcare providers; step 2–3 digital tools are not yet fully developed.
Xuan et al. (2025) (34)	China	Qualitative study	20/not specified	DHS-ICM	Routine monitoring, Comprehensive assessment, Cognitive game interaction, Machine learning prediction, Personalized care/goal plans, Health reminders, Abnormal alerts, Digital human interaction, Emotional support, Health management.	*Enabling factors*: improves accessibility and objectivity of monitoring; increases interest in and adherence to health management; reduces burden on healthcare providers; provides emotional support to alleviate loneliness in older adults. *Major barriers*: digital divide exists; concerns over data security and personal information leakage; some older adults perceive monitoring as a reminder of ageing, leading to resistance; may exacerbate health inequalities.
Woo et al. (2025) (33)	China	Descriptive study	3,300/approximately 5-month observation	iHealth Screen	Routine monitoring, Comprehensive assessment, Resource centre.	*Enabling factors*: download and usage data indicate its acceptance; qualitative feedback considers it an easy-to-use self-management tool. *Major barriers*: insufficient motivation for continued use among users; awareness of the value of standardized assessment tools needs improvement.
Gaussens et al. (2023) (30)	France	Cross-sectional study	14,572/not specified	ICOPE MONITOR mobile app	Comprehensive assessment, Abnormal alerts.	*Enabling factors*: the platform supports systematic screening for decline in intrinsic capacity in primary care. *Major barriers*: the continuity of monitoring is low.
Seidel et al. (2022) (36)	Pan-European	Mixed-methods study	>2000/4 years	SHAPES Platform	Routine monitoring, Data sharing hub, Machine learning prediction, Abnormal alerts, Needs identification, Personalized care/goal plans.	Not reported

Two platforms were designed to monitor four domains of intrinsic capacity (25, 26, 28, 29, 35), including locomotion, psychological capacity, vitality, and cognition. The SMART system (29) supports daily monitoring, comprehensive assessment, health management and reminders, and anomaly alerts. It further enables the collection and storage of user preferences to inform personalized care planning, as well as timely referral and follow-up. The ProACT platform (25, 26, 28, 35) shares similar core functionalities with the SMART system (29), but does not include comprehensive assessment or user preference acquisition and storage capabilities. Detailed information is provided in Table 2.

**Table 2:** Summary of digital health platform characteristics.

Author, year	Country	Study design	Sample size/intervention duration	Platform name	Core functions	Outcomes
Guo et al. (2025) (29)	China	Cross-sectional study	110/not specified	SMART System	Routine monitoring, Comprehensive assessment, Personalized care/goal plans, Health management, Collection/storage of user behavior/preferences, Health reminders, Abnormal alerts, Referral/follow‑up.	*Enabling factors*: high perceived usefulness; significant health benefits; high user control; *Major barriers*: low perceived ease of use; poor device compatibility
Dinsmore et al. (2021) (25)	Ireland, Belgium	Prospective cohort study	120/12 months	ProACT Platform	Routine monitoring, Data sharing hub, Personalized care/goal plans, Health reminders, Abnormal alerts, Referral/follow‑up.	*Enabling factors*: feasibility and ease of use; high user compliance; user reports of improved self-management skills, health, and well-being; and the importance placed on triage services *Major barriers*: frustration caused by unreliable technical equipment
Doyle et al. (2021) (26)	Ireland, Belgium	Prospective cohort study	120/12 months			*Enabling factors*: high user engagement, increased confidence and ability in self-management, increased physical activity, improved diet, and a platform that is accessible and easy to use *Major barriers*: a small number of dropouts attributed their withdrawal to technical issues with the devices, including Bluetooth connection and synchronization failures, missed or incorrect data entries, device power-on/off failures, and the complexity of the devices and apps, or an excessive health-related burden.
Sheng et al. (2024) (28)	Ireland	Longitudinal study	60/approximately 12 months			*Enabling factors*: over 80% of participants used the technological devices for more than 200 days, indicating a very high level of overall engagement. *Major barriers*: self-reported engagement and continuity were significantly lower than those automatically recorded by the devices.
Doyle et al. (2022) (35)	Ireland, Belgium	Qualitative study	120/approximately 1 year			*Enabling factors*: triage nurses provide clinical, educational, and emotional support, enhance patients’ self-management confidence, improve patients’ response speed to abnormal symptoms and adherence to medical advice, and reduce unnecessary use of medical resources. *Major barriers*: some patients experience monitoring anxiety.

The remaining three platforms covered more limited domains of intrinsic capacity. The inCASA system (37) focuses on vitality and locomotion, offering daily monitoring, data sharing, anomaly detection, and referral or follow-up functions. The e-Frail Navi platform (32) targets locomotion only and provides daily monitoring, alerting, and machine-learning–based prediction. The ProVista Care system (23) assesses locomotion, psychological capacity, and vitality, and in addition to daily monitoring, alerts, and health management and reminders, also enables comprehensive evaluation of medication use. Detailed characteristics are summarized in Table 3.

**Table 3:** Summary of digital health platform characteristics.

Author, year	Country	Study design	Sample size/intervention duration	Platform name	Core functions	Outcomes
Gokalp et al. (2018) (37)	UK	Observational study	36/8 months	inCASA Integrated Monitoring Platform	Routine monitoring, Abnormal alerts, Referral/follow‑up, Data sharing hub.	*Enabling factors:* The platform enables long‑term monitoring, with generally good patient compliance; abnormal vital sign data can trigger effective clinical interventions; changes in activity patterns are associated with events such as acute exacerbation of disease and falls. *Major barriers:* Small sample size; comfort and reliability issues with bed and chair sensors; the need to reduce false alarms.
Uenishi and Song (2025) (32)	Japan	Descriptive study	Not specified/not specified	e-Frail Navi	Routine monitoring, Machine learning prediction, Abnormal alerts.	*Enabling factors:* With an accuracy rate exceeding 80%, among 11 individuals diagnosed with frailty, 8 showed improvement in health status. *Major barriers:* Older adults are unable to view or understand the information.
Wong et al. (2024) (23)	China	Randomized controlled trial	76 (39 intervention, 37 control)/3 months	ProVista Care	Routine monitoring, Comprehensive assessment, Health management, Health reminders, Abnormal alerts.	*Enabling factors:* High adherence, high self-efficacy, reduced anxiety levels, high feasibility, improved perceived ease of use; the “Live With WMD Program” effectively enhanced older adults’ proficiency and confidence in using the device. *Major barriers:* Poor device reliability, compatibility issues, lack of user-centreed design.

#### 3.2.2 Usage models of digital health platforms

In terms of system architecture, most platforms adopted a multi-user, multi-device collaborative design. Seven platforms explicitly differentiated three primary user groups—older adults, informal or formal caregivers, and healthcare professionals—and provided dedicated applications or web-based portals for each group, thereby enabling data sharing and coordinated task management (24–27, 29, 31, 36). Three platforms distinguished two user groups, namely older adults and healthcare professionals (34, 35, 37). Two platforms were designed exclusively for healthcare professionals or staff (30, 32), while one platform targeted older adults only (28). One platform differentiated between older adults and care institutions (33). In addition, the ProVista Care platform featured a distinct tri-terminal configuration, supporting dedicated interfaces for older adults, community workers, and nurses (23).

#### 3.2.3 Data sources of digital health platforms

Data acquisition and monitoring capabilities constitute the foundational infrastructure of digital health platforms. A wide range of heterogeneous data sources were identified.

Wearable and mobile devices, such as smart bands and accelerometers, were commonly used for continuous monitoring of steps, activity levels, and heart rate, enabling assessment of vitality and locomotion capacity (24–26, 28, 29, 35–37). Environmental sensors, including passive infrared motion sensors, smart mattresses, and smart electricity meters, provided non-intrusive monitoring of daily activities and behavioral patterns, primarily for assessing locomotion capacity and detecting abnormal events such as fall risk (25, 26, 32, 35–37). Self-reported data, collected via in-platform electronic questionnaires, were widely used to assess multiple domains of intrinsic capacity—including cognition, psychological health, sensory function, and vitality—as well as symptoms and medication-related information (23–31, 33–36). Medical devices, such as Bluetooth-enabled blood pressure monitors and glucose meters, enabled the collection of specific physiological parameters (24, 28). Interaction and behavioral data were also captured, including cognitive game interaction logs via tablets and platform usage metadata (24, 26, 33). In addition, some systems integrated external data sources, such as electronic health records (36). Emerging data modalities included information derived from interactions with digital avatars and motion data analysed via computer vision techniques (34).

The integration of these multimodal data streams enables a complementary and multidimensional assessment of intrinsic capacity, spanning subjective self-reports and objective measurements, as well as intermittent evaluations and continuous real-world monitoring.

#### 3.2.4 Outcomes

##### 3.2.4.1 User acceptability and adherence

Multiple studies demonstrated that digital health platforms exhibited good feasibility and user acceptability among community-dwelling older adults. In terms of acceptability, four studies reported that the platforms were perceived as easy to use (23–25, 33). Regarding implementation outcomes, positive effects were primarily reflected in three domains. First, user engagement was generally high, with four studies reporting strong adherence (23, 25, 34, 37) and two studies indicating sustained participation levels (26, 28). Second, high levels of user satisfaction were reported (27). Third, in terms of objective performance, one study confirmed the accuracy of platform-based assessments (32).

##### 3.2.4.2 Health outcomes and self-management

Several platforms demonstrated potential benefits in improving health outcomes and self-management capabilities. Kolakowski et al. (24) reported that the platform contributed to fall prevention and improvements in cognitive function. Uenishi and Song (32) found that the e-Frail Navi system, leveraging smart meter data, led to improved health outcomes in 8 of 11 frail older adults. Wong et al. (23) observed that use of the ProVista Care platform enhanced self-efficacy and reduced anxiety among older adults. Both Doyle et al. (26) and Dinsmore et al. (25) reported that the ProACT platform strengthened users’ confidence and capacity for self-management, with improvements observed in physical activity levels and dietary behaviors. Xuan et al. (34) demonstrated that the DHS-ICM system increased older adults’ engagement and adherence to health management activities while also providing emotional support.

##### 3.2.4.3 Healthcare resource utilisation and timely intervention

Digital health platforms supported timely clinical intervention through remote monitoring and automated anomaly detection, thereby contributing to more efficient healthcare resource utilisation. The ICOPE platform developed by Tavassoli et al. (27, 31) enabled large-scale remote screening and timely intervention. The inCASA system developed by Gokalp et al. (37) facilitated clinically relevant interventions, with changes in activity patterns associated with disease-related events. Doyle et al. (35) demonstrated that nurse-led triage supported by the ProACT platform reduced unnecessary healthcare utilisation while improving patient treatment adherence. Gaussens et al. (30) reported that the ICOPE MONITOR application enabled systematic screening for frailty within primary care settings.

##### 3.2.4.4 Barriers and challenges

**Figure 2:**
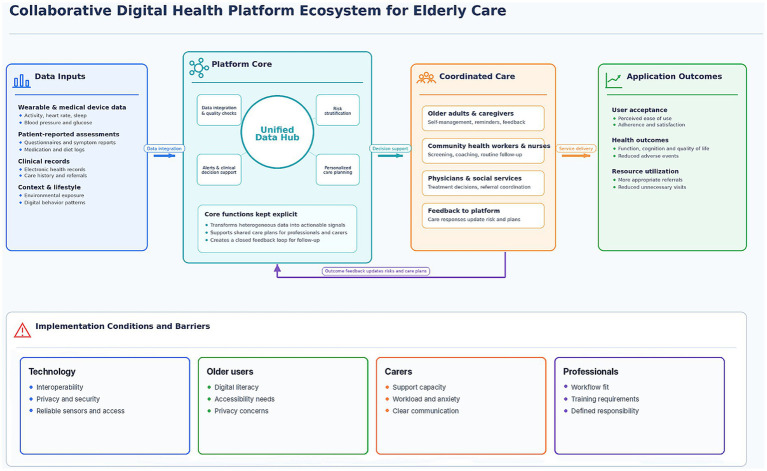
Overview of the implementation of digital health platforms.

Despite the demonstrated benefits of digital health platforms, several barriers to implementation were identified. Technical and device-related issues were frequently reported, including poor interoperability between devices and systems (29), Bluetooth connectivity failures (25, 26, 28), missing data entries (26), and limited comfort associated with wearable sensors (37). At the user level, multiple factors adversely affected engagement. These included the digital divide (24–26, 29, 34, 35), concerns regarding data security and privacy, resistance to continuous monitoring (34), potential financial burden associated with platform use (24), and a lack of sustained motivation for long-term engagement (33). In addition, monitoring-related anxiety was reported among some participants (35), and adherence to self-reported data collection was lower than that observed for automated sensor-based monitoring (28). Future work should prioritise improving technical robustness, strengthening user training and privacy protection, and accounting for individual heterogeneity in digital health adoption. The key characteristics and implementation determinants of the included digital health platforms are summarised in Figure 2.

## 4 Discussion

This review included 15 studies and synthesized the technological typologies, core functionalities, and application outcomes of digital health platforms. The findings indicate that integrated care models enabled by digital health platforms can support the assessment and dynamic monitoring of intrinsic capacity in older adults, with potential benefits in fall prevention, cognitive improvement, enhanced self-management skills, and reductions in anxiety and depressive symptoms (23, 24, 26). However, results across studies were heterogeneous and influenced by multiple contextual and system-level factors. Accordingly, the following discussion critically examines these determinants from four perspectives: older adult users, caregivers, healthcare professionals, and platform-related technical factors.

### 4.1 Older adults users

With advancing age, most older adults prefer to age in place within familiar community environments (38, 39). However, the integration of digital health technologies into community-based geriatric care or integrated home-care systems continues to face substantial challenges (40, 41). At present, many digital health platforms are primarily designed for monitoring rather than empowerment. Xuan et al. (34) reported that some older adults perceived continuous monitoring as a marker of frailty or decline, leading to resistance. Similarly, Doyle et al. (26) found that persistent monitoring may impose a substantial psychological burden on older users. These findings are fundamentally misaligned with the principles of the ICOPE framework, which emphasises early detection of intrinsic capacity decline to preserve functional autonomy; however, if monitoring is interpreted as signalling dependency, it may paradoxically undermine intended outcomes.

This study identified perceived usability as a key determinant of older adults’ acceptance of digital health technologies (42). Usability testing of the SMART system revealed relatively low scores in perceived ease of use, with system complexity and multiple functions leading some users to feel overwhelmed during learning and adoption (29). Consistent with the PeeK study (43), adoption of home-based ageing technologies is influenced by 27 factors, among which usability and privacy concerns are predominant. A plausible explanation is that many platforms are developed without sufficient understanding of older adults’ real-world needs, resulting in a mismatch between system functionality and user capabilities (44). Wegener et al. (45) further noted that frail older adults are often excluded from technology design processes, leading to underrepresentation of their needs, whereas participatory design approaches can significantly enhance acceptance and willingness to use such technologies. Notably, among the included platforms, only a small number explicitly reported user involvement in the design process (29), while most provided limited or no description of participatory design methodologies.

From the perspective of the ICOPE framework, coverage of the five domains of intrinsic capacity remains uneven across existing digital health platforms. Most systems focus on locomotion, psychological capacity, vitality, and cognition, whereas sensory function is markedly underrepresented, with only a limited number of platforms incorporating sensory assessments (24, 27, 30, 31, 33, 34, 36). However, declines in vision and hearing represent early indicators of functional deterioration in older adults and constitute core screening components recommended by ICOPE. This gap may reduce the sensitivity of digital health platforms in detecting early-stage declines in intrinsic capacity.

### 4.2 Caregiver perspectives

Digital health platforms are increasingly reshaping caregivers’ roles, shifting them from traditional burden-bearers to supportive partners in older adults’ health trajectories. The SMART system integrates informal caregivers into its service framework; through personalised interventions, it positions caregivers not merely as passive contributors, but as structured collaborators with clearly defined tasks and guidance (29). The ProACT platform enables older adults to actively invite members of their care network and provides them with dedicated applications, thereby supporting differentiated data-sharing arrangements (25, 26). These design features collectively reflect a growing recognition of the critical role of caregivers in integrated care ecosystems.

However, this review found that most platforms are primarily designed for older adults or healthcare professionals, with limited functionality tailored specifically to caregivers and a lack of dedicated support modules (24, 29). For caregivers—particularly those providing long-term care—high workload, prolonged caregiving demands, restricted daily routines, and reduced social participation may contribute to psychological distress, including anxiety and depression. Despite this, current platforms largely overlook caregivers’ emotional stress and psychological burden (24). Moreover, caregivers prioritise not technological sophistication per se, but whether platforms can effectively reduce caregiving burden and provide clear, actionable care guidance. At present, coordination, communication, and information-sharing mechanisms between formal and informal caregivers are often insufficiently addressed, potentially compromising care quality (25). Although integrated care is fundamentally grounded in person-centred, cross-role collaboration, exclusion of caregivers from digital care ecosystems undermines the integrity of the care continuum.

Therefore, while prioritising older adults’ health needs, it is equally essential to strengthen caregiver-oriented support modules, including emotional support, caregiving skills training, and stress management services. System design should explicitly incorporate caregiver needs to alleviate caregiving burden. Online support communities could be established to facilitate professional exchange, resource sharing, and peer support among caregivers (46–48). In terms of data sharing, older adults should retain autonomy over what information is disclosed, thereby ensuring privacy protection while enabling caregivers to access essential health information for informed care provision.

### 4.3 Health care provider perspectives

Digital health platforms provide healthcare professionals with remote monitoring capabilities and decision-support tools (49, 50). The CAREUP platform uses radar charts and time-series trend visualisations to deliver evidence-based information for the development of personalised intervention strategies (24). The SMART system includes a dedicated professional interface, enabling clinicians to review and adjust system-generated care plans, thereby improving transparency and interpretability in decision-making processes (29). The ICOPE programme established a remote monitoring infrastructure in which experienced geriatric nurses manage incoming alerts, substantially reducing the monitoring burden on frontline healthcare staff (27, 31). Similarly, triage nurses within the ProACT platform integrate data from multiple sources to construct comprehensive geriatric health profiles, enabling holistic assessment and timely intervention (35). Collectively, these systems are reshaping the role of healthcare professionals from passive responders to proactive care managers.

Nevertheless, limitations remain in the design and functionality of professional-facing interfaces. This review identified a persistent lack of system integration as a common challenge across platforms (51). Most systems operate independently of hospital electronic health record (EHR) systems, creating new information silos that hinder the integration of platform-generated data into routine clinical workflows (52). From a clinical perspective, simple threshold-based alert systems frequently generate a high rate of false positives, contributing to increased workload and alert fatigue among healthcare professionals (37). In addition, service models limited to weekday daytime operations reduce accessibility, leaving abnormal events occurring outside working hours insufficiently addressed (25, 35). These limitations are largely attributable to the fact that most existing platforms implement only the screening stage of the ICOPE five-step pathway, without effectively linking subsequent stages, thereby constraining the full realisation of integrated care. As highlighted by Gaussens et al. (30), the ICOPE MONITOR mobile application supports systematic screening in primary care settings; however, monitoring continuity remains limited, indicating that even when intrinsic capacity decline is detected, sustained intervention and follow-up may not be ensured, thereby reducing overall clinical efficiency.

To address these challenges, future platforms should strengthen decision-support functionalities for healthcare professionals and prioritise interoperability with hospital EHR systems in accordance with established standards to enable seamless data integration (36). Dedicated clinical dashboards should be developed to integrate alert rules, intervention recommendations, and evidence-based guidelines, alongside intelligent tiered alerting systems to minimise disruption from non-urgent notifications (24, 29, 37, 53). Furthermore, clear clinical protocols are required to standardise the use of predictive outputs and define liability frameworks to ensure patient safety. In addition, evidence from the ICOPE programme suggests that embedding integrated care pathways within reimbursement systems and providing financial incentives to participating healthcare professionals are critical factors for improving engagement and implementation sustainability (54).

### 4.4 Technology platforms

Digital health platforms demonstrate an increasingly diversified technological landscape. These systems commonly adopt multi-role, multi-device architectures, with dedicated applications developed for older adults, caregivers, and healthcare professionals, respectively, (24, 25, 36). Data acquisition has become progressively more heterogeneous, integrating wearable devices, environmental sensors, medical devices, user-generated inputs, avatar-based interactions, and computer vision–enabled analytics (24, 34). Core functionalities typically include daily monitoring, anomaly detection, comprehensive assessment, personalised care planning, and health information delivery (24, 29). More advanced platforms further incorporate machine-learning–based predictive models to estimate future trajectories of functional capacity (24), or employ digital avatars to enable multimodal interaction and provide emotional support (34).

However, several technical challenges remain (55–57). Limited interoperability across systems and devices is a pervasive issue. For example, the SMART system is currently compatible only with Android smartphones and does not support iOS devices (29), thereby excluding a substantial proportion of older adults who use Apple devices. Such compatibility constraints not only restrict platform reach but may also exacerbate inequities in access to digital health resources. Although some systems monitor all five domains of intrinsic capacity, predictive stability—particularly for the psychological domain—remains limited (24). The five domains defined by intrinsic capacity are not equally amenable to measurement; psychological capacity, in particular, is strongly influenced by subjective factors, making real-time and fully reliable prediction challenging. In addition, financial burden represents a major barrier. The CAREUP platform, for instance, involves an initial cost of €325 and a monthly fee of €7.50 (24), which may constitute a substantial economic constraint for older adults with limited financial resources. For individuals with low income, those living alone, or those relying on fixed pensions, such additional expenses may directly lead to discontinuation of use. Although some platforms, such as iHealth Screen, are publicly available at no cost (33), the long-term sustainability of free-access models and their capacity to support continuous maintenance and updates remain uncertain.

To address these challenges, there is a need to develop trustworthy, open, and sustainable digital health platforms (58, 59). Cross-platform compatibility for both Android and iOS should be prioritised (33), alongside simplified versions tailored for older adults with limited digital literacy. For intrinsic capacity monitoring, domains characterised by high variability and strong subjectivity may be better suited to periodic, standardised assessments rather than continuous real-time prediction. Collaboration with health insurance systems or the adoption of tiered pricing models could be explored to subsidise or fully cover costs for economically disadvantaged older adults (60). In parallel, partnerships with device manufacturers should be strengthened to enhance system stability and optimise user experience through user-centred design principles (61–63). Evidence from the SHAPES project suggests that the development of open, extensible digital health ecosystems that enable third-party innovation is a critical pathway for ensuring long-term platform sustainability (34).

## 5 Limitations

This study has several limitations. First, only English-language publications were included, which may have led to the underrepresentation of relevant studies conducted in non-English-speaking countries, particularly in rapidly developing ageing-care contexts such as China and Japan. Although both British and American spelling variants were used in the search strategy, a substantial number of North American studies may have been excluded during initial screening due to non-compliance with predefined eligibility criteria rather than search sensitivity.

Second, the geographic distribution of the included studies was uneven, with 67% originating from Europe and 33% from Asia. This overrepresentation of high-income regions limits the generalisability of the findings, particularly for low- and middle-income countries, where digital health infrastructure, resource availability, and care delivery models may differ substantially.

In light of these limitations, future research should expand geographic coverage, with particular emphasis on generating empirical evidence from low- and middle-income settings. In addition, greater efforts are needed to translate study protocols into full-scale trial reports, thereby strengthening the evidence base on intervention effectiveness and enhancing the global applicability of digital health solutions for older adults.

## 6 Conclusion

This systematic review provides a comprehensive synthesis of the existing evidence on the monitoring capabilities of digital health platforms in integrated care for community-dwelling older adults. The findings suggest that such models hold substantial potential for supporting proactive health management, enhancing self-management capacity, and optimising healthcare resource allocation. However, reported effects remain heterogeneous, and successful implementation depends on coordinated engagement across four key domains: older adults, caregivers, healthcare professionals, and technological systems.

Older adults need to bridge the digital divide and transition from passive monitoring recipients to active participants in their own health management. Caregivers can be repositioned from implicit burden-bearers to collaborative partners in care delivery. Healthcare professionals must address challenges related to inadequate system integration and increased workload. From a technological perspective, there is a need to develop trustworthy, open, and sustainable digital health platforms. Future research should prioritise co-design approaches, stratified training programmes, enhanced system interoperability, standardisation efforts, and supportive policy frameworks.

## Data Availability

The original contributions presented in the study are included in the article/Supplementary material, further inquiries can be directed to the corresponding author.
